# Barriers to help-seeking for postpartum depression mapped onto the socio-ecological model and recommendations to address barriers

**DOI:** 10.3389/fgwh.2024.1335437

**Published:** 2024-05-24

**Authors:** Jean Marie S. Place, Kalyn Renbarger, Kristin Van De Griend, Maya Guinn, Chelsie Wheatley, Olivia Holmes

**Affiliations:** ^1^ Department of Nutrition and Health Science, Ball State University, Muncie, IN, United States; ^2^ School of Nursing, Ball State University, Muncie, IN, United States; ^3^ Department of Community and Public Health, Idaho State University, Pocatello, ID, United States; ^4^ Department of Biology, Ball State University, Muncie, IN, United States; ^5^ Medical Imaging, Idaho State University, Pocatello, ID, United States

**Keywords:** postpartum depression, help-seeking, barriers, socio-ecological model, perinatal mood and anxiety disorder (PMAD)

## Abstract

Postpartum depression affects nearly a quarter of women up to a year after childbirth. Although it is treatable, significant barriers to help-seeking prevent women from being treated. This paper assesses key literature on the barriers for help-seeking among women with postpartum depression. The barriers identified have been mapped onto the socio-ecological model in addition to potential recommendations that professionals can use to address barriers on individual, interpersonal, organizational, community and societal levels. The recommendations provided are meant to serve as leverage points for professionals in efforts to create appropriate support and interventions. As such, this paper serves as a mapping tool for healthcare and public health professionals to assess obstacles to women's help-seeking and to guide multi-pronged interventions on various levels of the socio-ecological model that may increase help-seeking among women with postpartum depression. Holistically and comprehensively providing support to women will require significant effort throughout all sectors of society as opposed to isolated, siloed interventions.

## Introduction

Depression during the postpartum period (PPD) is classified as major depression with onset in the first four weeks postpartum (1), or up to one year after delivery, with symptoms that last for at least two weeks (2). Postpartum depression affects up to a quarter of women up to a year after childbirth (3), with people of color experiencing substantially higher risk compared to Caucasian women (4). Compared to the “baby blues,” which is common, mild, emotional volatility lasting for several weeks after delivery, the duration of PPD can last for several months. Symptoms include depressed or angry feelings, withdrawal from loved ones, feelings of being numb or disconnected, intrusive, worrisome thoughts about hurting the baby (without intent to do so), feelings of guilt about not being a good parent, or harboring doubts about one's ability to care for the baby. Such symptoms interfere with activities of daily living, decrease a woman's quality of life, and negatively impact parent-child bonding (5–8).

Postpartum depression is highly treatable (9). Antidepressant medication, psychotherapy, counseling, and interpersonal therapy have all been shown to be effective treatments (10). Despite the various options, postpartum depression is the most underdiagnosed pregnancy-related health problem in the U.S. (6, 11), with a significant proportion of women not receiving treatment.

In a landmark article, Dennis and Chung-Lee (12) systematically assessed PPD help-seeking barriers and maternal treatment preferences, which had not been done previously. They reviewed peer-reviewed publications to catalog and qualitatively categorize help-seeking barriers for PPD. Updated research in the form of a critical commentary by Grissette et al. (13) confirmed many of Dennis and Chung-Lee's original findings, with results suggesting women with PPD face multiple roadblocks that impede women's access to treatment. We have identified and included several additional barriers by reviewing relevant, recently published literature. Taken together, we classify selected barriers according to the different levels of the socio-ecological model (SEM). Our mapping process involved assigning three reviewers to individually extract barriers that Dennis and Chung-Lee's and Grissette et al. outlined in their work and classify unique barriers according to a level of the SEM. Discrepancies by reviewers were resolved by discussion among all authors. We include practical recommendations for professionals in efforts to create appropriate support and interventions at multiple levels and we emphasize the importance of taking a holistic perspective and working not only within but across levels to provide more comprehensive support to women.

**Figure 1:**
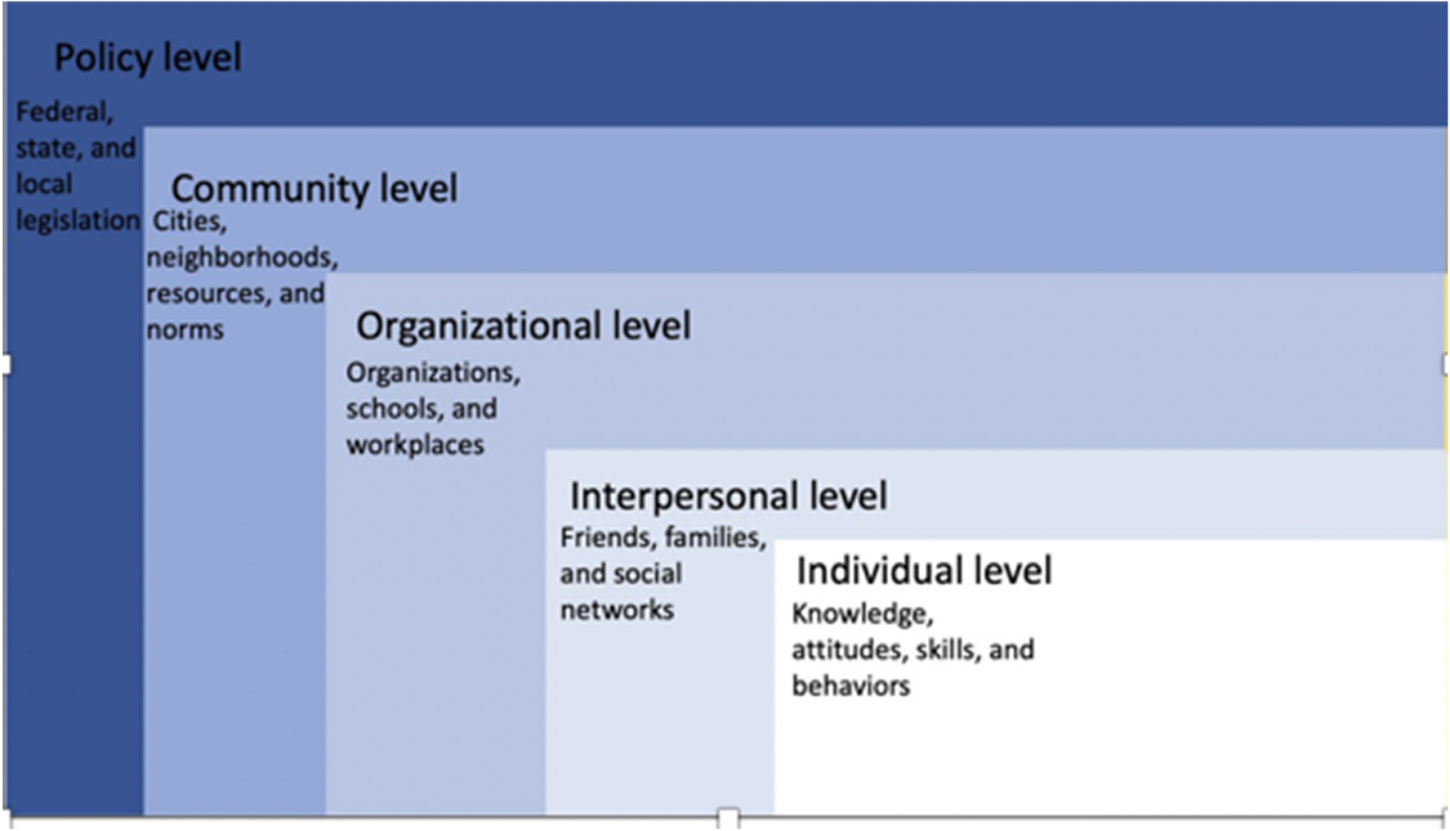
Depiction of the layers of the socio-ecological model (SEM).

The SEM is a theory-based framework useful to understand the multifaceted personal and environmental influences on behavior (14). The SEM offers a comprehensive perspective on health by considering various interconnected levels: individual, interpersonal, organizational, community, and policy (see Figure 1). It comprises five systems, emphasizing two personal factors (individual, interpersonal) and three outer factors (community, organizational, and societal), with the personal factors exerting the most significant influence. These systems collectively shape the environment, which the SEM argues has a significant impact on developmental outcomes. In the context of PPD, the SEM provides a framework for examining influences on help-seeking. The purpose of mapping the barriers and facilitators of women's help-seeking onto a theoretical framework is two-fold: First, the SEM encourages an expanded view on women's utilization of services by moving beyond personal factors that may limit or enable help-seeking to broader interpersonal, institutional, cultural, and political influences. Second, conceptualizing barriers for women's help-seeking behavior from the perspective of a theoretical framework assists professionals to understand and focus on the areas in which they are positioned to intervene in efforts to create improved access to and acceptability of care, and to collaborate efficiently on implementing multi-level interventions. The purpose of this paper is to map previous research findings and recommendations onto the SEM to guide healthcare and public health professionals to implement interventions that increase help-seeking among women with PPD (Table 1).

**Table 1:** Barriers and recommendations for help-seeking for PPD, mapped onto the socio-ecological model.

Level of the socio-ecological model	Description of level on the socio-ecological model	Barriers to PPD Help-seeking	Recommendations to address Barriers
Individual level	An individual's demographic information has the capacity to influence health outcomes, including an individual's gender, religion, race, ethnicity, age, socioeconomic status, and sexual orientation. Additionally, an individual's attitudes, beliefs, behaviors, and level of knowledge shape their health status.	• Detrimental beliefs on help-seeking • Lack of knowledge • Resistance to pharmaceutical treatments • Time management concerns	• Awareness campaigns • Social media toolkits • Warmlines and hotlines • Faith-based support • Patient decision-making aids • Telehealth and text-messaging services
Interpersonal level	An individual's formal and informal relationships are determining factors of health status. These may include family, peer, and patient-provider relationships.	• Negative views or low awareness of PPD among family & friends • Healthcare providers who lack warmth or dismiss symptoms	• Online support forums for moms and dads • Peer mentoring programs • Group prenatal care
Organizational/institutional level	Institutions have the power to shape individuals’ behaviors and attitudes, thereby contributing to health status, due to organizational characteristics, regulations, rules, operations, and cultural expectations within the institution.	• Lack of culturally competent care and language support services • Lack of time and/or privacy with provider • Lack of healthcare accessibility, referral protocol, and follow-up • Inadequate Medicaid coverage	• Screenings for and awareness of risk factors for perinatal mood & anxiety disorders during pregnancy and the postpartum • Perinatal psychiatric consultation services • Utilization of community health workers • Provision of informational and emotional support for infant care • Practice readiness assessments, such as PREPD
Community/structural level	The environment around individuals influences health behavior and health status. The community environment includes norms, access to resources, and the built environment, including buildings, parks, greenspace, and city-wide infrastructure.	• Unsupportive community norms or cultural models of maternal role • Lack of transportation • Lack of childcare • Workforce shortages • Shame, embarrassment, and stigma	• Community events (e.g., Climb Out of the Darkness walks) • Recognition of World Maternal Mental Health Day • Co-location of mental health and medical services • Culturally diverse, peer support groups (online or in-person)
Societal/public policy level	Laws, policies, and allocation of funding are instrumental in determining health outcomes.	• Inadequate insurance coverage • Immigration status • Poverty	• Comprehensive reimbursement systems • Medicaid expansion • Enhanced screening and referral services • Affordable and accessible childcare

### Individual-level barriers and recommendations for help-seeking

Individual-level barriers of help-seeking for PPD in Dennis and Chung-Lee's (12) and Grissette et al.'s (13) work, as well as in additional literature (15), indicate that there is a strong belief among some women that needing help makes someone weak, and many women have a strong sense of self, preventing them from believing they need care or seeking care (16, 17). Other women report being unaware that symptoms of PPD could be classified as problematic, with some women lacking knowledge on where and how to access resources and treatment options (12, 13). Awareness campaigns, such as the Blue Dot Project that is now incorporated as an international awareness symbol through Postpartum Support International, are an important access point to connect individuals and families to resources and support. The Blue Dot Project (18) is the official host of the Maternal Mental Health awareness week. Other social media toolkits that promote inclusive, knowledge-building of signs, symptoms, resources, and support are important, especially in spaces occupied by women and new mothers.

Women in the perinatal period have reported resistance to pharmaceutical treatments, including taking antidepressants due to fear of addiction, side effects to self or baby through breast-milk transmission, or perception of long-term harms (12, 13). These concerns are particularly salient for Black mothers (18). Because women reported feeling an insufficient knowledge base to make decisions regarding pharmaceutical treatment, patient decision-making aids may be useful in providing guidance in clinically complex cases. Research has indicated high patient satisfaction with such aids (19).

Finally, time management is reported as an individual-level barrier to help-seeking for PPD. Women have reported insufficient time to attend appointments, due to scheduling conflicts or a perception that other demands on their time are more important. If women fear contacting the wrong person when they reach out to traditional medical establishments, they are less likely to disclose symptoms. Telehealth services can ease concerns about time, reducing wait times and the need for in-person follow-up care (20, 21). Telehealth modalities include video conferencing, mobile health, and text messaging that can be used with mental health counseling and health promotion interventions that do not require a physical presence but provide face-to-face communication. Text messaging can also be used for appointment reminders and motivational messaging (22, 23).

### Interpersonal-level barriers and recommendations for help-seeking

Interpersonal barriers in Chung-Lee's (12) and Grissette et al.'s (13) work, as well as other literature (15), include negative views of PPD from partners, family and friends. Gender identity, racial identity, and class may make some women uniquely susceptible to facing discrimination from others, which may increase the likelihood and severity of PPD symptoms and negatively impact help-seeking (24). A lack of emotionally warm and empathetic support can also be detrimental to seeking mental health treatment. Studies indicated there is a low awareness among partners and other family members about perinatal mood disorders like PPD and the treatment of them, as well as a lack of knowledge on how to support someone with a disorder (25). This lack of knowledge and support can increase the negative stigma associated with PPD (26), and is directly linked to symptoms of PPD (27). One way to promote support is families, partners, friends, and providers being aware of online forums in which women can participate. Online forums can provide social support for new mothers, facilitate the sharing of information, act as a source of entertainment, and provide a sense of community for women who might otherwise feel isolated due to the demands of new motherhood (28). *Postpartum Support International* offers women and men opportunities to connect with other moms and dads, respectively, in online support groups where the first 30 min is for providing information, education, and establishing group guidelines. The next 60 min is considered “talk time,” in which women and men can share and talk with each other. *Postpartum Support International* also offers a Peer Mentor Program. This program pairs individuals in need of support with a trained volunteer who has also experienced and fully recovered from a perinatal mood disorder. The goal of the program is to offer encouragement and hope from someone who has experienced a perinatal mood disorder by establishing a one-to-one connection with an individual who is struggling (29).

Additional barriers to help-seeking include health professionals who minimize and dismiss depressive symptoms, force the medicalization of depression through pharmaceutical treatment, show disinterest, display patronizing attitudes, or do not engage in discussions about PPD and other perinatal mood disorders (12, 13). Health care providers such as childbirth educators, midwives, and nurses are in key positions to make personal connections with women, offer emotional support, and make recommendations and referrals to community resources to help women enjoy the postpartum experience (11). Strong, close relationships between the healthcare provider and the patient facilitate help-seeking for PPD, particularly when disclosure is met with understanding, recognition of the problem, and reassurances that they are not alone (12, 13). Providers can also boost women's perception of social support by offering group prenatal care. Group prenatal care, as opposed to traditional models of prenatal care where women receive individual health care services from clinical providers during pregnancy, has been shown to increase women's perception of social support, boost relationships with providers, and improve their emotional well-being (30). Furthermore, having positive relationships with providers and peers through group prenatal care can promote a supportive environment to address PPD and facilitate help-seeking behaviors (31).

### Organizational/institutional-level barriers and recommendations for help-seeking

Organizational/institutional-level barriers in Dennis and Chung-Lee's (12) and Grissette et al.'s (13) work, as well as other literature (15), indicate that a lack of physicians from minority backgrounds and language barriers inhibit help-seeking, underscoring the importance of culturally competent care and language support services. Due to cultural differences, questionnaires for perinatal mood disorders need to be specifically tailored to language, country of origin, or culture to have accurate diagnostic information (32). In a feasibility study measuring the effectiveness of a Telemedicine Perinatal Mental Health Model among Latina women in Southern California, Baker-Ericzén (33) et al. found that mothers who participated in a culturally sensitive mental health program reported higher rates of satisfaction and more access to community care. Baker-Ericzén et al. (34) found that providing culturally-sensitive care on perinatal mood disorders, and connecting the importance of screening/treatment of perinatal mood disorders to cultural values is essential. Allowing access to providers who speak the same language is also important for women experiencing depression to describe their experiences in their native language.

Other barriers include a lack of time and/or privacy with the provider, which contributes to perceptions that thorough mental health assessments are not conducted or that exams are insufficient, thus dissuading women from help-seeking or follow-up care. When providers are competent in following standards of care, consumer confidence increases and may underscore help-seeking behaviors (35). For example, the ACOG (34) recommends women who have current depression or anxiety, a history of perinatal mood disorders, risk factors for perinatal mood disorders, or suicidal thoughts should receive close monitoring, evaluation, and assessment. Nurses, in particular, play an important role in identifying women with perinatal mood disorders. They need to know how to aid in screening for perinatal mood disorders, be well-versed in the options for care and interventions, as well as possible barriers that their patients may be facing (36). Knowing the risk factors for PPD can help facilitate appropriate triage. Risk factors include depression or anxiety during pregnancy, experiencing stressful life events during pregnancy or the early postpartum period, traumatic birth experience, preterm birth/infant admission to neonatal intensive care, low levels of social support, previous history of depression, and breastfeeding problems (34, 37).

Health care professionals should follow the ACOG recommendations that all obstetric care providers complete an assessment for depression and anxiety at least once in the perinatal period. The American Academy of Pediatrics recommends expanding screening through infant's 1, 2, 4, and 6-month well child visits (38). The ACOG also recommends that providers complete a full assessment of mood and emotional well-being during the comprehensive postpartum visit using a validated instrument. One such validated instrument for PPD and anxiety is the Edinburgh Postnatal Depression Scale (EPDS) and is the most frequently used in research and clinical practice settings (39). The EPDS consists of 10 self-reported questions that take less than 5 min to complete. Real-time perinatal psychiatric consult lines should be available to providers with patients who screen positive for psychosis, suicidality, or thoughts of harming a child (40).

In addition to self-assessment tools, it is important to give pregnant women the pertinent information and education on postpartum changes and infant care so that they feel more equipped with their new role and lifestyle, as well as the difficulties that may come up due to it. Providers who give informational and emotional support to pregnant women about infant-care have been shown to decrease the number of women who had postpartum anxiety and improved the safety and welfare of infants (41). Community health workers are key partners in the health system and can be trained to screen and refer women to mental health services, provide support, and accompany them to healthcare visits (42).

Other barriers include long wait lists to be seen by a provider, difficulty navigating medical phone trees, early discharge after delivery, and a lack of referral protocol or provider follow-up. Because initiation of treatment or referral to mental health care providers offers maximum treatment benefits, the ACOG recommends that clinical staff in obstetrics and gynecology practices should be prepared to initiate medical therapy and refer women to behavioral health resources when needed (2). To increase the confidence and competence of providers, and potentially have a positive downstream effect on women's help-seeking behaviors, healthcare providers are encouraged to complete a practice readiness assessment, such as the Practice Readiness to Evaluate and address Perinatal Depression (PREPD), to inform the development of policies and procedures in implementing perinatal depression screening as standard care (43). Additionally, lack of medical providers who accept Medicaid is a salient organizational barrier that prevents women from receiving the care and screenings they need. State-level Medicaid expansion has been shown to increase the number of providers who accept Medicaid (44).

### Community/structural-level barriers and recommendations for help-seeking

Community/structural-level barriers in Dennis and Chung-Lee's (12) and Grissette et al.'s (13) work, as well as other literature (15), indicate community norms and cultural models of the maternal role and mental health affect women's help-seeking. Women of ethnic minorities have reported wanting the opportunity to meet other mothers in similar situations either in group settings or in community-based peer support groups (45). The use of online support groups can provide cultural support for women experiencing PPD. *Postpartum Support International* offers many culturally diverse, online support groups including groups for queer and trans parents, Black parents, Latinx parents, single parents, and military mothers.

Researchers have documented that women often feel self-shame or embarrassment when needing outside help for PPD, and they have a negative stigma associated with the disclosure of this information (15, 16, 46, 47). These community-level barriers are particularly salient for women of color, low-income women, or among those with lower levels of health literacy (16, 17, 48, 49). Warmlines or hotlines for PPD support can be an important source of support for women who may be reluctant to access traditional treatment or face other barriers to care. Telephone support has shown to decrease postpartum depressive symptomology (50). Telephone support is offered in multiple languages by *Postpartum Support International* and the HRSA-operated National Maternal Mental Health Hotline. Additionally, faith-based institutions may be able to reach women in non-traditional spaces and enhance protective factors such as social support. *Postpartum Progress* has a list of potential sources of support according to different religions (51). Finally, programs and events to promote awareness of PPD are important to empower women to seek treatment and receive the support they need. *Postpartum Support International* hosts community events to bring awareness of the issues surrounding PPD. One example, the Climb Out of the Darkness community walk, is an international fundraiser where survivors, providers, and members of the community come together to promote awareness for PPD (52). Community members, health care providers, and other maternal mental health advocates can bring awareness of PPD by signing a petition calling on the World Health Assembly and the UN World Health Organization to officially recognize World Maternal Mental Health Day, to be commemorated annually on the first Wednesday of May (53).

Barriers in the built environment include lack of personal transportation and lack of childcare (12). Additionally, women who live in rural communities and states have greater disadvantages than their urban counterparts and are at a much higher risk of experiencing PPD throughout their lifetime (54). They may have a lower socioeconomic status than women in urban areas, which can make it harder to get to a facility to be screened and cared for (55, 56). This is due, in part, to the increasing closures of critical access hospitals and the workforce shortages that are especially common in rural communities (57, 58). Mestad et al. (59) suggest co-locating multiple health services to help at-risk populations with new motherhood. One example of innovative funding is the Health Resources and Services Administration (HRSA) funding for Screening and Treatment for Maternal Depression and Related Behavioral Disorders. Through this program, behavioral health and care coordination has been integrated into maternal healthcare through a real-time consult line (60). Healthcare providers have also been trained on perinatal mood and anxiety disorders, equipping more providers with relevant skills (3, 59).

The presence of peer support groups with childcare services in communities can reduce logistical barriers and serve as an access point for women to receive help for symptoms of PPD. Some in-person programs consist of a free, peer-support group, developed to increase social support and destigmatize postpartum mood symptoms. Groups can be held weekly and co-facilitated by former group attendees and maternal health professionals (29).

### Societal-level/public policy barriers and recommendations for help-seeking

Societal-level barriers in Dennis and Chung-Lee's (12) and Grissette et al.'s (13) work, as well as other literature, indicate that lack of access to healthcare is a primary barrier to women's help-seeking, particularly if a woman is uninsured, underinsured, or at risk of exposure to immigration enforcement due to immigration status. Economic barriers, poverty, and mental health shortage areas, especially in rural areas, are all known barriers to PPD treatment (26). Advocacy efforts for policies and funding that support maternal health is critical. Comprehensive reimbursement systems, statewide Medicaid expansion (61, 62), and support for community-based health services, including Head Start (63), Healthy Start, home visitation programs (39), Title V, and Block Grants that provide screening and referral services for PPD can improve maternal mental health outcomes and reduce disparities (64). Paid leave and making childcare available and affordable may reduce the impact of PPD on the child (65, 66), reduce the risk of PPD (67), and allow women to seek help for PPD.

## Conclusion

Postpartum depression is a multi-faceted problem, influenced by diverse forces in society. As such, it is important to elucidate the facilitators and barriers for help-seeking on multiple levels of the socio-ecological model so a multi-faceted and multi-pronged approach to prevention, detection and treatment can be advanced. Because many barriers are not isolated on a singular level, but rather are compounded by interactions and influences from various levels of the SEM, understanding how recommendations can be broached and interwoven throughout the individual, interpersonal, community, and societal levels can contribute to more comprehensive, easily accessible, and appropriate care (68). For example, barriers on an individual level such as detrimental beliefs regarding help-seeking may be intensified by family and friends’ views of PPD or healthcare providers who are not trained in competent, compassionate care. Barriers on an organizational level, such as lack of time and/or privacy with a provider may be intensified by community norms of shame and stigma around mental health. Barriers are ultimately more challenging in situations of poverty or without adequate legal or health insurance protections.

The entry points for intervention on an individual-level include promotion of awareness campaigns and social media toolkits, as well as other tailored tools for women to gain information and support, such as warm lines, hot-lines, and decision-making aids. The entry points for intervention on an interpersonal level include promoting resources to increase peer support, building relationships of trust between providers and patients, and working to address stigma associated with PPD. The entry points for intervention on an organizational level include promotion of self-report assessments and screening tools, as well as front-line provider education. Other options include working with providers who are not embedded in the traditional health system, such as community health workers and peer-support volunteers. The entry points for intervention on a community level include co-locating services that can address multiple needs of mothers simultaneously, community awareness events, such as the worldwide Climb Out of the Darkness walk, sponsored by PSI International, and actively working toward culturally-responsive services with a racially and linguistically diverse workforce. Finally, the entry points for intervention on a societal level include policy proposals such as paid leave, affordable childcare and Medicaid expansion. The more healthcare and public health professionals work to implement interventions across multiple levels of the SEM, the greater the potential impact of enhancing women's help-seeking for PPD.

Some limitations to our mapping tool exist. It was difficult to limit each barrier to only one level of the SEM when, in reality, they could be placed in multiple levels. Future research should be conducted to address gaps related to the intersectionality of factors such as race, ethnicity, gender, and socio-economic status. Additionally, there is a lack of research on the role of fathers in help-seeking for PPD. More research which is inclusive of fathers is needed to address barriers and facilitators to help-seeking for PPD.

## Data Availability

The original contributions presented in the study are included in the article, further inquiries can be directed to the corresponding author.

## References

[1] American Psychiatric Association. Depressive Disorders: DSM-5® Selections. Washington DC, United States: American Psychiatric Association Publishing (2016).

[2] American College of Obstetricians and Gynecologists Committee. Treatment and management of mental health conditions during pregnancy and postpartum: aCOG clinical practice guideline No. 5. Obstet Gynecol. (2023) 141(6):1262–88. 10.1097/AOG.0000000000005202 37486661

[3] Bauman BL Ko JY Cox S D'Angelo D Warner L Folger S Vital signs: postpartum depressive symptoms and provider discussions about perinatal depression—United States, 2018. MMWR Morb Mortal Wkly Rep. (2020) 69:575–81. 10.15585/mmwr.mm6919a2 32407302 PMC7238954

[4] Black KA MacDonald I Chambers T Ospina MB. Postpartum mental health disorders in indigenous women: a systematic review and meta-analysis. J Obstet Gynaecol Can. (2019) 41(10):1470–8. 10.1016/j.jogc.2019.02.009 30981617

[5] Brooks E Cox E Kimmel M Ruminjo A. Risk of untreated symptoms of PMADs in pregnancy and lactation. In Cox E. editor, Women’s Mood Disorders: A Clinician’s Guide to Perinatal Psychiatry. Cham, Switzerland: Springer (2021). p. 45–53.

[6] Drake E Howard E Kinsey E. Online screening and referral for postpartum depression: an exploratory study. Community Ment Health J. (2013) 50(3):305–11. 10.1007/s10597-012-9573-3 23283485 PMC3646921

[7] Flynn HA. Epidemiology and phenomenology of postpartum mood disorders. Psychiatr Ann. (2005) 35(7):544–51. 10.3928/0048-5713-20050701-12

[8] Sutter-Dallay AL Giaconne-Marcesche V Glatigny-Dallay E Verdoux H. Women with anxiety disorders during pregnancy are at increased risk of intense postnatal depressive symptoms: a prospective survey of the MATQUID cohort. Eur Psychiatry. (2004) 19(8):459–63. 10.1016/j.eurpsy.2004.09.025 15589703

[9] Miller LJ. Postpartum depression. JAMA. (2002) 287(6):762–5. 10.1001/jama.287.6.762 11851544

[10] Fitelson E Kim S Baker A Leight K. Treatment of postpartum depression: clinical, psychological and pharmacological options. Int J Womens Health. (2011) 3:1–14. 10.2147/IJWH.S6938 PMC3039003 21339932

[11] Corrigan CP Kwasky AN Groh CJ. Social support, postpartum depression, and professional assistance: a survey of mothers in the midwestern United States. J Perinat Educ. (2015) 24(1):48–60. 10.1891/1058-1243.24.1.48 26937161 PMC4720860

[12] Dennis CL Chung-Lee L. Postpartum depression help-seeking barriers and maternal treatment preferences: a qualitative systematic review. Birth. (2006) 33(4):323–31. 10.1111/j.1523-536X.2006.00130.x 17150072

[13] Grissette B Spratling R Aycock D. Barriers to help-seeking behavior among women with postpartum depression. J Obstetr Gynecol Neonatal Nurs. (2018) 47(6):812–9. 10.1016/j.jogn.2018.09.006 30296405

[14] McLeroy KR Bibeau D Steckler A Glanz K. An ecological perspective on health promotion programs. Health Educ Q. (1988) 15(4):351–77. 10.1177/109019818801500401 3068205

[15] Canty H Sauter A Zuckerman K Cobian C Grisby T. Mothers’ perspectives on follow-up for postpartum depression screening in primary care. J Dev BehavPediatr. (2019) 40:20. 10.1097/DBP.0000000000000628 30422838

[16] Cacciola E Psouni E. Insecure attachment and other help-seeking barriers among women depressed postpartum. Int J of Environ Res Public Health. (2020) 17(11):3887. 10.3390/ijerph17113887 32486285 PMC7313466

[17] Swami V. Mental health literacy of maternal and paternal postnatal (postpartum) depression in British adults. J. Ment Health. (2020) 29(2):217–24. 10.1080/09638237.2019.1608932 31070064

[18] MMH Awareness Week 2019—TheBlueDotProject Maternal Mental Health. TheBlueDotProject. Available online at: https://www.thebluedotproject.org/mmhweek2019 (Accessed April 01, 2024).

[19] Khalifeh H Molyneaux E Brauer R Vigod S Howard LM. Patient decision aids for antidepressant use in pregnancy: a pilot randomised controlled trial in the UK. BJGP Open. (2019) 3(4). 10.3399/bjgpopen19X101666 31822489 PMC6995861

[20] Gately ME Trudeau SA Moo LR. Feasibility of telehealth delivered home safety evaluations for caregivers of clients with dementia. OTJR (Thorofare, N.J.). (2020) 40(1):42–9. https://doi.org/ 10.1177/1539449219859935 31319745

[21] Kruse CS Krowski N Rodriguez B Tran L Vela J Brooks M. Telehealth and patient satisfaction: a systematic review and narrative analysis. BMJ Open. (2017) 7(8):e016242. 10.1136/bmjopen-2017-016242 28775188 PMC5629741

[22] Mayer JE Fontelo P. Meta-analysis on the effect of text message reminders for HIV-related compliance. AIDS Care. (2016) 29(4):409–17. 10.1080/09540121.2016.1214674 27477580 PMC5480218

[23] Saberi P Dawson Rose C Wootton AR Ming K Legnitto D Jeske M Use of technology for delivery of mental health and substance use services to youth living with HIV: a mixed-methods perspective. AIDS Care. (2019) 32(8):931–9. 10.1080/09540121.2019.1622637 31132864 PMC6881543

[24] Floyd James K Smith B Robinson M Tobin C Bulles K Barkin J. Factors associated with postpartum maternal functioning in black women: a secondary analysis. J Clin Med. (2023) 12(2):647. 10.3390/jcm12020647 36675575 PMC9862142

[25] Negron R Martin A Almog M Balbierz A Howell EA. Social support during the postpartum period: mothers’ views on needs, expectations, and mobilization of support. Matern Child Health J. (2013) 17(4):616–23. 10.1007/s10995-012-1037-4 22581378 PMC3518627

[26] Alfayumi-Zeadna S Froimovici M Azbarga Z Grotto I Daoud N. Barriers to postpartum depression treatment among Indigenous Bedouin women in Israel: a focus group study. Health Soc Care Community. (2018) 27(3):757–66. 10.1111/hsc.12693 30488992

[27] Bayrampour H McDonald S Tough S. Risk factors of transient and persistent anxiety during pregnancy. Midwifery. (2015) 31(6):582–9. 10.1016/j.midw.2015.02.009 25823754

[28] Teaford D McNiesh S Goyal D. New mothers' experiences with online postpartum forums. MCN Am J Matern Child Nurs. (2019) 44(1):40–5. 10.1097/NMC.0000000000000489 30444739

[29] Prevatt BS Lowder EM Desmarais SL. Peer-support intervention for postpartum depression: participant satisfaction and program effectiveness. Midwifery. (2018) 64:38–47. 10.1016/j.midw.2018.05.009 29908406

[30] Renbarger KM Place JM Schreiner M. The influence of four constructs of social support on pregnancy experiences in group prenatal care. Womens Health Reps. (2021) 2(1):154–62. 10.1089/whr.2020.0113 PMC8243703 34235502

[31] Heberlein E Picklesimer A Billings D Covington-Kolb S Farber N Frongillo E. The comparative effects of group prenatal care on psychosocial outcomes. Arch Womens Ment Health. (2015) 19:295–69. 10.1007/s00737-015-0564-6 26260037

[32] Arifin SRM Cheyne H Maxwell M. Review of the prevalence of postnatal depression across cultures. AIMS Public Health. (2018) 5(3):260–95. 10.3934/publichealth.2018.3.260 30280116 PMC6141558

[33] Baker-Ericzén MJ Connelly CD Hazen AL Dueñas C Landsverk JA Horwitz SM. A collaborative care telemedicine intervention to overcome treatment barriers for Latina women with depression during the perinatal period. Fam Syst Health. (2012) 30(3):224–40. 10.1037/a0028750 22709321 PMC3780578

[34] American College of Obstetricians and Gynecologists Committee. Opinion No. 757: screening for perinatal depression. Obstet Gynecol. (2018) 132(5):e208–12. 10.1097/AOG.0000000000002927 30629567

[35] McGuire A White D Bartholomew T Flanagan M McGrew J Rollins A The relationship between provider competence, content exposure, and consumer outcomes in illness management and recovery programs. Adm Policy Ment Health. (2017) 44(1):81–91. 10.1007/s10488-015-0701-6 26563769

[36] Arefadib N Shafiei T Cooklin A. Barriers and facilitators to supporting women with postnatal depression and anxiety: a qualitative study of maternal and child health nurses’ experiences. J Clin Nurs. (2022) 3:3–4. 10.1111/jocn.16252 PMC10078709 35156748

[37] Robertson E Grace S Wallington T Stewart DE. Antenatal risk factors for postpartum depression: a synthesis of recent literature. Gen Hosp Psychiatry. (2004) 26(4):289–95. 10.1016/j.genhosppsych.2004.02.006 15234824

[38] Lamere K Golova N. Screening for postpartum depression during infant well child visits: a retrospective chart review. Clin Pediatr (Phila). (2022) 61(10):699–706. 10.1177/00099228221097272 35588233

[39] Levis B Negeri Z Sun Y Benedetti A Thombs B. Accuracy of the Edinburgh postnatal depression scale (EPDS) for screening to detect major depression among pregnant and postpartum women: systematic review and meta-analysis of individual participant data. BMJ. (2020) 371:m4022. 10.1136/bmj.m4022 33177069 PMC7656313

[40] Langan R Goodbred A. Identification and management of peripartum depression. Am Fam Physician. (2016) 93(10):852–8. 27175720

[41] Hijazi HH Alyahya MS Al Abdi RM Alolayyan MN Sindiani AM Raffee LA The impact of perceived social support during pregnancy on postpartum infant-focused anxieties: a prospective cohort study of mothers in Northern Jordan. Int J Womens Health. (2021) 13:973–89. 10.2147/IJWH.S329487 34707417 PMC8544270

[42] Boyd RC Mogul M Newman D Coyne JC. Screening and referral for postpartum depression among low-income women: a qualitative perspective from community health workers. Depress Res and Treat. (2011) 2011:1–7. 10.1155/2011/320605 PMC3096153 21603131

[43] Masters GA Brenckle L Sankaran P Moore Simas TA Person SD Allison J Development of the practice readiness to evaluate and address perinatal depression (PREPD) assessment. Psychiatry Res. (2021) 302:114032. 10.1016/j.psychres.2021.114032 34111739 PMC8277728

[44] Ortega A. Medicaid expansion and mental health treatment: evidence from affordable care act. Health Econ. (2023) 32(4):755–806. 10.1002/hec.4633 36480355

[45] Watson H Harrop D Walton E Young A Soltani H. A systematic review of ethnic minority women’s experiences of perinatal mental health conditions and services in Europe. PLoS One. (2019) 14(1):e0210587. 10.1371/journal.pone.0210587 30695019 PMC6351025

[46] Bodnar-Deren S Benn E Balbierz A Howell E. Stigma and postpartum depression treatment acceptability among black and white women in the first six-months postpartum. Matern Child Health J. (2017) 21(7):1457–68. 10.1007/s10995-017-2263-6 28102504

[47] Jones A. Help seeking in the perinatal period: a review of barriers and facilitators. Soc Work Public Health. (2019) 34(7):596–605. 10.1080/19371918.2019.1635947 31242074

[48] Kozhimannil K Trinacty C Busch A Huskamp H Adams A. Racial and ethnic disparities in postpartum depression care among low-income women. Psychiatr Serv. (2011) 62(2):619–25. 10.1176/ps.62.6.pss6206_0619 21632730 PMC3733216

[49] Bina R Glasser S. Factors associated with attitudes toward seeking mental health treatment postpartum. Women Health. (2018) 59(1):1–12. 10.1080/03630242.2017.1421286 29281589

[50] Dennis CL Kingston D. A systematic review of telephone support for women during pregnancy and the early postpartum period. J Obstet Gynecol Neonatal Nurs. (2008) 37(3):301–14. 10.1111/j.1552-6909.2008.00235.x 18507601

[51] Stone K. Postpartum Depression Resources for Different Religious Faiths. POSTPARTUM PROGRESS. (2010). Available online at: https://postpartumprogress.com/postpartum-depression-resources-christian-jewish-morm on-religious (Accessed April 01, 2024).

[52] Postpartum Support International (PSI). (2023). Available online at: https://www.postpartum.net/join-us/climbout/ (Accessed April 01, 2024).

[53] WMMH Day—World Maternal Mental Health awareness day [Internet]. WMMH Day. Available online at: https://wmmhday.postpartum.net/#:∼:text=World%20Maternal%20Mental%20Health%20Day%20%2D%203%20May%202023 (Accessed April 01, 2024).

[54] Mollard E Hudson D Ford A Pullen C. An integrative review of postpartum depression in rural U.S. Communities. Arch Psychiatr Nurs. (2016) 30(3):418–24. 10.1016/j.apnu.2015.12.003 27256951

[55] Luca DL Margiotta C Staatz C Garlow E Christensen A Zivin K. Financial toll of untreated perinatal mood and anxiety disorders among 2017 births in the United States. Am J Public Health. (2020) 110(6):888–96. 10.2105/AJPH.2020.305619 32298167 PMC7204436

[56] Nidey N Tabb KM Carter KD Bao W Strathearn L Rohlman DS Rurality and risk of perinatal depression among women in the United States. J Rural Health. (2019) 36(1):9–16. 10.1111/jrh.12401 31602705

[57] Bai G Yehia F Chen W Anderson GF. Varying trends in the financial viability of US rural hospitals, 2011–17. Health Aff. (2020) 39(6):942–8. 10.1377/hlthaff.2019.01545 32479226

[58] Kaufman BG Thomas SR Randolph RK Perry JR Thompson KW Holmes GM The rising rate of rural hospital closures. J Rural Health. (2015) 32(1):35–43. 10.1111/jrh.12128 26171848

[59] Mestad R Lane SD Hall M Smith CJ Carter DB Rubinstein RA Prenatal depression: screening and referral for women who are low income during antenatal care. Soc Work Public Health. (2016) 31(6):557–64. 10.1080/19371918.2016.1160344 27286463

[60] Screening and Treatment for Maternal Depression and Related Behavioral Disorders Program (MDRBD) | MCHB. mchb.hrsa.gov. Health Resources & Services Administration; Available online at: https://mchb.hrsa.gov/programs-impact/screening-treatment-maternal-depression-related-behavioral-disorders-program-mdrbd (Accessed April 01, 2024).

[61] Krohn J Matone M. Supporting mothers with mental illness: postpartum mental health service linkage as a matter of public health and child welfare policy. PubMed. (2017) 30(1):1–19. 30889321

[62] Margerison CE Hettinger K Kaestner R Goldman-Mellor S Gartner D. Medicaid expansion associated with some improvements in perinatal mental health. Health Aff. (2021) 40(10):1605–11. 10.1377/hlthaff.2021.00776 PMC9007181 34606358

[63] Lee K Hunter T. The associations between maternal depressive symptoms and parenting practices among low income head start eligible families. Soc Work Ment Health. (2021) 20(2):203–25. 10.1080/15332985.2021.1999365

[64] Bigby J Anthony J Hsu R Fiorentini C Rosenbach M. Recommendations for maternal health and infant health quality improvement in Medicaid and the Children’s Health Insurance Program (2020). Available online at: https://www.medicaid.gov/medicaid/quality-of-care/downloads/mih-expert-workgroup-recommendations.pdf (Accessed April 01, 2024).

[65] Mandal B. The effect of paid leave on maternal mental health. Matern Child Health J. (2018) 22(10):1470–6. 10.1007/s10995-018-2542-x 29882033

[66] Herba CM Tremblay RE Boivin M Liu X Mongeau C Séguin JR Maternal depressive symptoms and children’s emotional problems: can early child care help children of depressed mothers? JAMA Psychiatry. 2013;70(8):830–8. 10.1001/jamapsychiatry.2013.1361 23784556

[67] Johnson AD Padilla CM. Childcare instability and maternal depressive symptoms: exploring new avenues for supporting maternal mental health. Acad Pediatr. (2019) 19(1):18–26. 10.1016/j.acap.2018.05.006 29852269

[68] Eriksson M Ghazinour M Hammarström A. Different uses of bronfenbrenner’s ecological theory in public mental health research: what is their value for guiding public mental health policy and practice? Soc Theory Health. (2018) 16:414–33. 10.1057/s41285-018-0065-6

